# The synthesis of green fluorescent carbon dots for warm white LEDs†

**DOI:** 10.1039/c8ra02226g

**Published:** 2018-05-29

**Authors:** Qin Du, Jingxia Zheng, Junli Wang, Yongzhen Yang, Xuguang Liu

**Affiliations:** Key Laboratory of Interface Science and Engineering in Advanced Materials (Taiyuan University of Technology), Ministry of Education Taiyuan 030024 China liuxuguang@tyut.edu.cn yyztyut@126.com; College of Chemistry and Chemical Engineering, Taiyuan University of Technology Taiyuan 030024 China; Research Center of Advanced Materials Science and Technology, Taiyuan University of Technology Taiyuan 030024 China; College of Materials Science and Engineering, Taiyuan University of Technology Taiyuan 030024 China

## Abstract

Green fluorescent carbon dots (CDs) were synthesized with pyrogallic acid as carbon source by solvothermal method in *N*,*N*-dimethylformamide (DMF). During the formation of the CDs, DMF not only serves as solvent for reaction, but also as nitrogen source to participate in the reaction. At the same time, it promotes the formation of large conjugated sp^2^-domain in CDs. The prepared CDs have an average size of 11.9 nm, excitation-independent emission centered at 520 nm and fluorescence quantum yield of 16.8%. For practical applications, warm white light-emitting diodes were fabricated by combining the CDs/*N*-[3-(trimethoxysilyl)propyl]ethylenediamine (KH-792) mixture with UV chip, which emitted warm white light with color coordinates of (0.39, 0.47) and a correlated color temperature of 4323 K suitable for indoor lighting.

## Abstract 2

Green emissive carbon dots synthesized by solvothermal method is used for warm white light-emitting devices.

## Graphical abstract

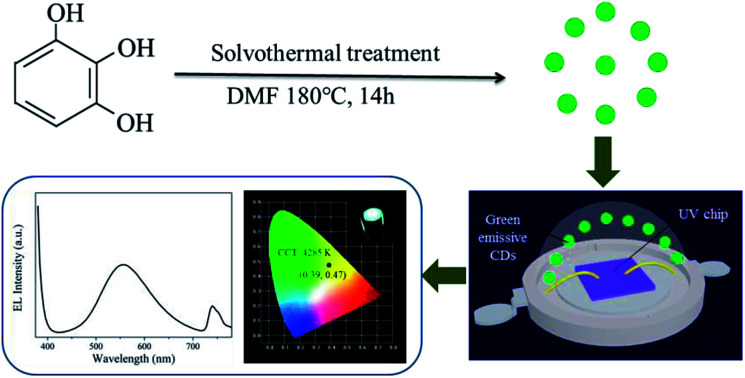

## 1 Introduction

Light-emitting diodes (LEDs) are fourth generation solid-state lighting sources after incandescent, fluorescent and high-intensity discharge lamps owing to their long service lifetime, low energy consumption and excellent environmental friendliness. Carbon dots (CDs), as a promising LED phosphor with the advantages of green, low cost, good stability and excellent optical properties,^1–4^ have broad application prospects in optoelectronic devices.

The application of CDs in LED has been reported so far. However, the correlated color temperature of the prepared white LED is over 5000 K, which belongs to the category of cool white light and is not suitable for occasions needing warm white such as indoor lighting, since most of prepared CDs emit blue light with maximal emission wavelength around 440 nm.^5–7^ Guo and co-workers^8^ synthesized poly(styrene-glycidyl methacrylate) and pyrolyzed the polymer at 200, 300 and 400 °C to give blue, orange and white emissive CDs, respectively, for the preparation of warm white LED. Although multicolor CDs can be prepared simply by adjusting pyrolysis temperature, the complicated synthesis process of the precursor restricts their further application. Therefore, it is of urgent importance to develop a simple and facile method to prepare long wavelength fluorescent CDs with emission wavelength centered at 500 nm or more for the development of warm white LED for indoor lighting.

So far, much work has been carried out on spectral regulation of CDs, especially in terms of particle size, surface state/composition and structure. Huang *et al.*^9^ used sucrose as carbon source and phosphoric acid as dopant to hydrothermally synthesize CDs of three different particle sizes by adjusting the concentration of phosphoric acid, which emit blue, green and yellow fluorescence separately under UV excitation. By adjusting the concentration of phosphoric acid the size of the CDs was tuned and the fluorescence of CDs thus regulated. Furthermore, the yellow emissive CDs were oxidized or reduced to reach blue-shifted emission, which is different from previous report.^10^ Obviously, both particle size and surface state of CDs have important impact on fluorescence, while the mechanism of how the surface state influences the fluorescence is not clear. Li *et al.*^11^ used graphite rods as carbon source to produce CDs by alkali-assisted electrochemistry. Four kinds of CDs with different particle sizes were isolated from raw CDs by column chromatography. Their results showed that with the increase of particle size, the CDs emit blue, green, yellow and red fluorescence separately.

In addition to regulating the particle size of CDs, modifying the surface of CDs or altering the composition of CDs by heteroatom doping can also alter the fluorescence of CDs. Han *et al.*^12^ prepared blue fluorescent CDs from glucose and ethylene glycol by hydrothermal method, and modified the CDs with polyethyleneimine (PEI) as modifier. Though the emission wavelength of the CDs shows limited extent of redshift, the emission still belongs to the category of blue fluorescence. Hu *et al.*^13^ obtained carboxyl-rich CDs using citric acid as carbon source by solvothermal method and then modified the CDs with *p*-phenylenediamine, sulfanilic acid and 4-aminothiophenol as surface modifier to realize blue, cyan and yellow emission, respectively. It can be seen that the surface modification of CDs is an effective method to regulate the fluorescence of CDs. Liu *et al.*^14^ prepared blue and yellow emissive CDs by microwave-assisted hydrothermal method, with glutaraldehyde as carbon source and PEI as nitrogen source through adjusting the ratio of the two raw materials. The yellow emissive CDs have a higher degree of surface oxidation than the blue emissive CDs. The red-shift in emission wavelength is due to the introduction of more surface defects upon higher surface oxidation.

The fluorescence of CDs can also be regulated by adjusting the structure of CDs. Jiang *et al.*^15^ reported blue, green and red emissive CDs prepared from *m*-, *o*- and *p*-phenylenediamine through solvothermal method, respectively. The different PL characteristics of the three kinds of CDs was ascribed to the difference in particle size and nitrogen content tentatively. However, the influence of the internal structure of the three kinds of CDs on the fluorescence was not elucidated. Miao *et al.*^16^ reported a synthesis of multiple-color-emission CDs through controlling the extent of graphitization. Wang *et al.*^17^ used 1,3-dihydroxynaphthalene as carbon source and KIO_4_ as oxidant to obtain red emissive CDs by solvothermal method with ethanol as solvent. The degree of sp^2^ conjugation of the obtained CDs is higher than that of graphene dots. The red emission of the CDs is attributed to the large sp^2^-domain.

According to the works above, it is found that although the regulation mechanism of CDs fluorescence is not well elucidated, large amount of evidence^18–22^ has shown that, large particle size, large conjugated sp^2^-domain, high nitrogen-doping would contribute to long wavelength fluorescence emission of CDs.

In this article, solvothermal method is selected to prepare CDs owing to its advantages of easy operation, high safety, high purity and good dispersibility of the prepared product. In solvothermal methods, dimethylformamide (DMF) as high-boiling nonaqueous solvent is conducive to dehydration and carbonization of raw materials, which would facilitate the formation of CDs with large conjugated sp^2^-domain.^23^ Pyrogallic acid, with benzene structure and rich hydroxyl groups, would result in the CDs with large conjugated sp^2^-domain by dehydration and carbonization.^17^ Therefore, in present study, green emissive CDs were fabricated by solvothermal method with pyrogallic acid as carbon source and DMF as solvent and further used as phosphors for warm white LED preparation.

## 2 Experimental

### 2.1 Materials

Pyrogallic acid and *N*,*N*-dimethylformamide (DMF) were purchased from Kaitong Chemical Reagent Co., Ltd (Tianjin, China). *N*-(3-(Trimethoxysilyl) propyl)ethylenediamine (KH-792) was offered by Guangfu Technology Development Co., Ltd (Tianjin, China). All the chemicals were used as received without any further purification.

### 2.2 Synthesis of CDs

**Fig. 1:**
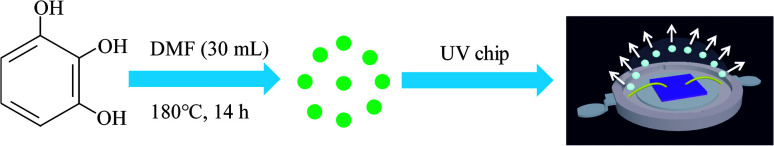
A schematic of CDs synthesis and their application in white LED.

Pyrogallic acid (0.4 g) and DMF (30 mL) were added into a 90 mL Teflon-lined stainless-steel autoclave and heated at different temperature (140, 160, 180, 200, 220 °C) for different reaction time (8, 10, 12, 14, 16 h). The schematic representation of the formation of CDs is illustrated in Fig. 1. After cooling to room temperature, the resulting brown-black solution was filtered by syringe filter (pore size: 0.22 mm) to remove larger carbon nanoparticles. Then the CDs were further dialyzed in a dialysis bag (cut-off molecular mass: 1000 Da). After 36 h the products from inside and outside dialysis bag were collected and stored for further use.

In consideration that DMF can help CDs form large conjugated sp^2^-domain and contribute to the green fluorescence emission of CDs, CDs-w and CDs-e were prepared under the same conditions with water and ethanol as solvents respectively.

### 2.3 Fabrication of warm white LEDs

For the fabrication of WLEDs based on nitrogen-doped CDs (N-CDs), UV chip with the peak wavelength centered at 365 nm was selected. CDs powder (5 mg) was dissolved in 10 mL of the mixture of KH-792 and deionized water (volume ratio of 1 : 1) under sonication for 3 min to form a homogenous solution. The homogenous solution (30 μL) was dropped into the conventional cup-shaped optical lens and thermally cured at 80 °C for 3 h. After that, the optical lens was capped on the bottom of the LED chip.

### 2.4 Characterization

High resolution transmission electron microscopy (HRTEM; JEM-2010, JEOL) was employed to characterize the morphology and size of CDs. The optical properties of CDs were measured by ultraviolet-visible spectrophotometry (UV-vis; U-3900, HITACHI) and photoluminescence spectrophotometry (PL; FluoroMax-4, HORIBA). Fourier transform infrared spectroscopy (FT-IR; TENSOR27, BRUKER) and X-ray photoelectron spectroscopy (XPS; Amicus Budget, SHIMADZU) were used to analyze surface functional groups of CDs. The elemental composition of the samples was determined by elemental analysis (EA; vario EL cube, ELEMENTAR). Transient fluorescence & phosphorescence (FLS980, EDINBURGH) was adopted in measuring the fluorescence decay status and further evaluating the fluorescence lifetime of CDs. The structure of CDs was characterized using Raman spectrometry (Raman; LabRAM HR Evolution) with radiation at 325 nm. The X-ray diffraction (XRD) patterns of CDs were recorded on a Rigaku-D/MAX 2500 diffractometer equipped with graphite monochromatized Cu Kα (*λ* = 1.54 Å) radiation at a scanning speed of 4° min^−1^ in the 2*θ* range from 10° to 90°. The thermal analysis (TA) of CDs was conducted using a NETZSCH TG209F3 instrument in nitrogen atmosphere at a heating rate of 10 °C min^−1^. A Spectra Scan PR 655 was used to analyze the emission spectra, CIE coordinates and CCT of white LEDs.

The quantum yield (QY) of CDs was determined by a relative method. Specially, rhodamine 6G (QY = 95% in ethanol, excitation wavelength 420 nm) was selected as the reference.^15^ A series of solutions of CDs and reference were prepared with concentrations adjusted such that the optical absorbance values were between 0–0.1 at 420 nm. The PL spectra were measured and the PL intensity was integrated. The integrated emission intensity is the area under the photoluminescence curve in the wavelength range 440–700 nm. The slope can be calculated from the plot of integrated emission intensity against absorbance. The QY of CDs was then calculated according to the following equation:
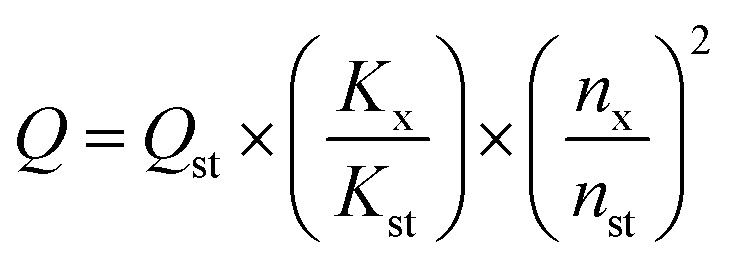
where *Q* is the QY of testing sample, *n* is the refractive index (1.43 for DMF and 1.36 for ethanol). The subscripts “st” and “x” refer to the reference and test sample, respectively.

## 3 Results and discussion

### 3.1 Optimization of the synthesis process of high QY CDs

**Fig. 2:**
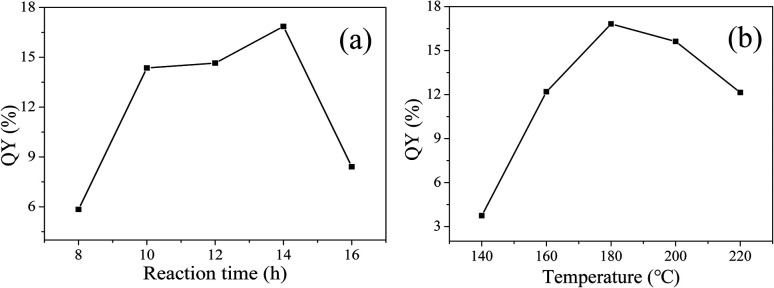
QY varies with reaction time (a) and temperature (b).

In order to prepare CDs with high QY, solvothermal reaction time and temperature were optimized. As shown in Fig. 2(a), at a reaction temperature of 180 °C, QY gradually increases with the increase of reaction time, maximizes at reaction time of 14 h, and then decreases. Therefore, 14 h is chosen as the optimal reaction time.

Then, the reaction time is fixed at 14 h, the influence of reaction temperature on QY is shown in Fig. 2(b). With the increase of reaction temperature, QY first increases and then decreases. The QY reaches the maximum of 16.82% at the reaction temperature of 180 °C. A reasonable explanation is that low temperature leads to insufficient carbonization, while high temperature is prone to give rise to severe carbonization, particle growth and aggregation, which are not favorable for fluorescence.^24^ So, the reaction time of 14 h and reaction temperature of 180 °C are selected as the optimum reaction conditions for the solvothermal synthesis of CDs.

### 3.2 Morphology and structure of original CDs (CDs-o)

**Fig. 3:**
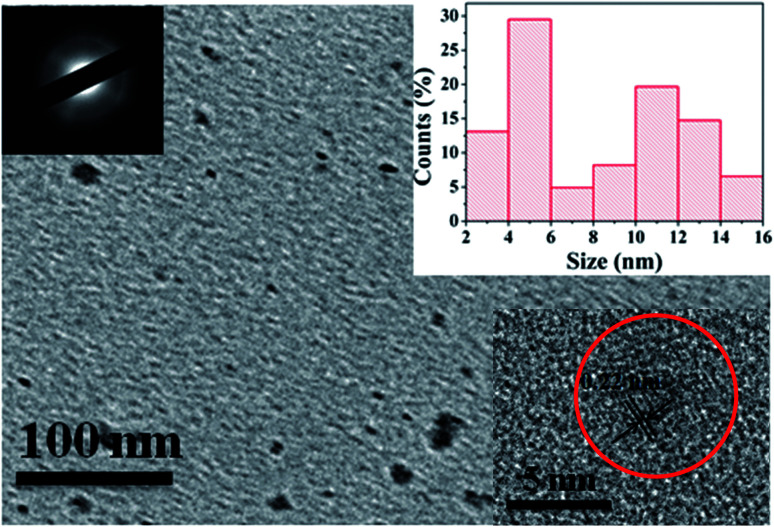
TEM image of CDs (inset is the SAED pattern, the size distribution image and HRTEM images of CDs).

To investigate the morphology and size of as-prepared samples, TEM observation was conducted. As shown in Fig. 3, original CDs are quasispherical and dispersed in broad size distribution falling into small size CDs (CDs-s) less than 10 nm and big size CDs (CDs-b) larger than 10 nm.

**Fig. 4:**
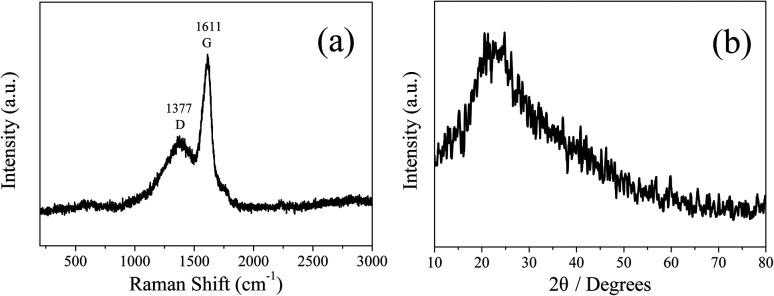
Raman spectrum (a) and XRD patterns (b) of CDs-o.

In order to analyze the structure of CDs-o further, Raman, XRD and SAED tests were performed. As shown in Fig. 4(a), Raman spectra of CDs-o show obvious characteristic D band at 1377 cm^−1^ and G band at 1611 cm^−1^. Remarkably, the intensity ratio *I*_G_/*I*_D_ is about 1.7, which confirms large conjugated sp^2^-domain in green emissive CDs-o,^17,25^ as will discussed below. Meanwhile, the XRD patterns of CDs-o display a broad diffraction peak centered at 2*θ* = 24.0°(Fig. 4(b)), corresponding to the moderate crystalline structure of CDs-o.^26^ Furthermore, HRTEM image (inset of Fig. 3) shows obvious lattice fringes, which confirms the crystalline feature of as-synthesized CDs. The lattice spacing is calculated to be 0.22 nm, which corresponds to the *d*-spacing of graphene {1–100} planes. In combination with the diffraction ring appearing in the SAED pattern (inset, Fig. 3), it can be confirmed that CDs-o are carbon nanoparticles with a moderate degree of crystallinity.

### 3.3 The surface structure of CDs-o

**Fig. 5:**
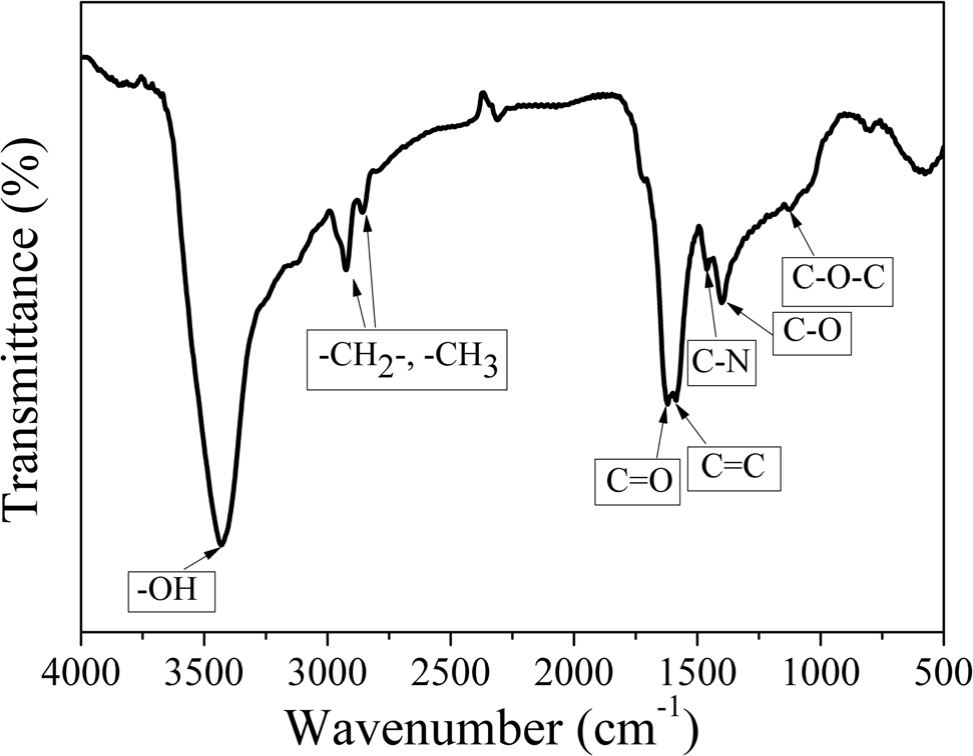
FT-IR spectra of the CDs-o.

FT-IR measurements were carried out to characterize the functional groups on CDs-o. As shown in Fig. 5, the distinct band around 3429 cm^−1^ can be ascribed to the stretching vibrations of O–H on the surface of CDs-o. The bands at 2926 and 2858 cm^−1^ represent the stretching vibrations of –CH_3_ and –CH_2_– in CDs-o. The obvious absorption band around 1620 cm^−1^ can be ascribed to the stretching vibrations of C=O, indicating that carboxyl groups exist on the surface of CDs-o. The absorption band around 1585 cm^−1^ is attributed to the vibration of C=C. The absorption bands around 1400 and 1126 cm^−1^ are attributed to C–O and C–O–C on the surface of CDs-o, indicating that the surface of CDs-o is rich in oxygen-containing functional groups, which endows CDs-o with good water solubility. The absorption band centered at 1462 cm^−1^ is attributed to the vibration of C–N, indicating that DMF not only acts as solvent providing a place for reaction, but also participates in the formation of CDs-o. The introduction of N atoms, as will be further confirmed later by elemental analysis, is beneficial to increasing the electron cloud density on the surface of CDs-o and facilitating the emission of long-wavelength fluorescence.^27^

**Fig. 6:**
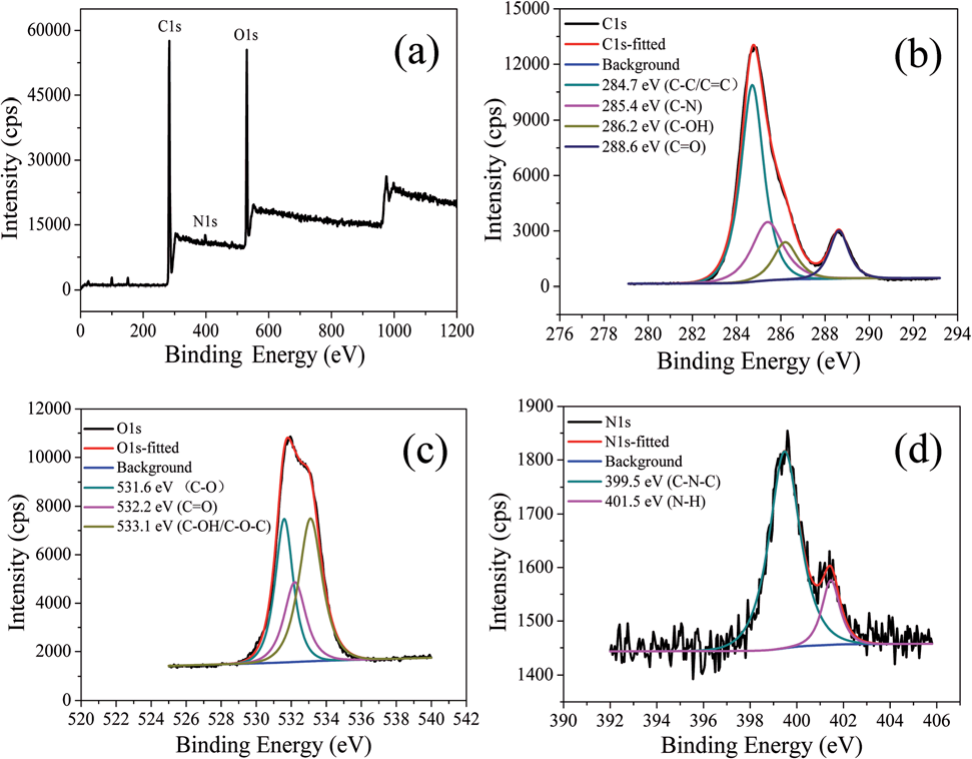
(a) XPS spectra of CDs-o. High resolution XPS spectrum of (b) C1s, (c) O1s, (d) N1s.

The chemical structure of CDs-o was probed using XPS. The wide scan XPS spectra of CDs-o are depicted in Fig. 6(a), where the strong O1s and C1s peaks demonstrate that CDs-o are principally composed of carbon and oxygen, while the relatively weak N1s peak further proves DMF participation in the formation of CDs-o. In the high-resolution XPS spectra, as Fig. 6(b) is shows, the C1s band can be deconvoluted into four peaks, corresponding to C=C/C–C (284.7 eV), C–N (285.4 eV), and C–OH (286.2 eV), C=O (288.6 eV). The O1s spectra (Fig. 6(c)) for CDs-o show that oxygen in CDs-o exists in three different chemical environments, corresponding to C–O (531.6 eV), C=O (532.2 eV) and C–OH/C–O–C (533.1 eV). Two types of N-related bonds are observed, including C–N–C (399.5 eV) and N–H (401.5 eV) (Fig. 6(d)).

### 3.4 Optical properties of green emissive CDs-o

**Fig. 7:**
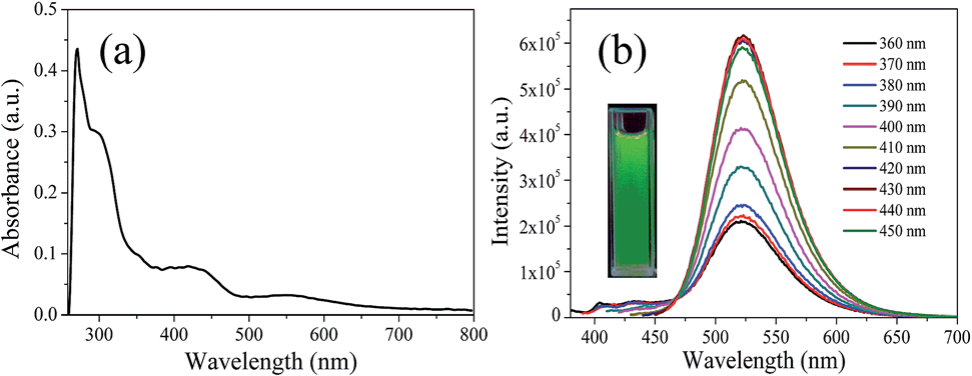
(a) UV-vis absorption spectrum, (b) PL spectra at different excitation wavelengths, inset: photograph of CDs-o under UV light.

In order to explore the fluorescence properties of CDs-o, UV-vis absorption and fluorescence spectra of CDs-o were tested. In the UV-vis absorption spectra (Fig. 7(a)), two obvious peaks, including a strong absorption characteristic peak at 270 nm and a relatively weak absorption peak at 310 nm, are observed, which are ascribed to π–π* transitions of C=C and n–π* transition of C=O bonds,^28^ respectively. What is more, an obvious visible absorption band peaked at 420 nm and a relatively weak absorption band centered at 540 nm can be seen, indicating large sized conjugated sp^2^-domain in the particles, which is the basis of long wavelength emission over 500 nm.^23,29,30^

The photoluminescence (PL), excited with a wide range of wavelengths, of CDs-o is shown in Fig. 7(b). As is shown in inset, CDs-o emit bright green fluorescence under the illumination of a hand-held UV lamp (365 nm). The emission peak of CDs-o solutions shows no shift and remains at 520 nm when the excitation wavelength changes from 360 to 450 nm. The photoluminescence wavelength of CDs-o is excitation-independent and the florescence intensity reaches the maximum with excitation at 420 nm. The QY of CDs-o is calculated to be 16.8%, higher than that (8.6%, 1.6%, 2.4%, 10.1%, 16.5%) for most reported CDs.^31–35^

**Fig. 8:**
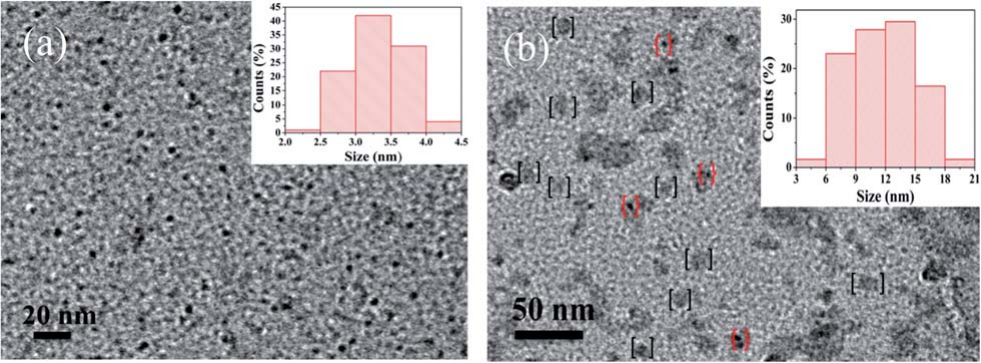
TEM images of CDs-s (a) and CDs-b (b) (inset is the size distribution, the dots in [ ] and { } refer to large sized CDs and small sized CDs aggregated to the surface of large sized CDs respectively).

**Fig. 9:**
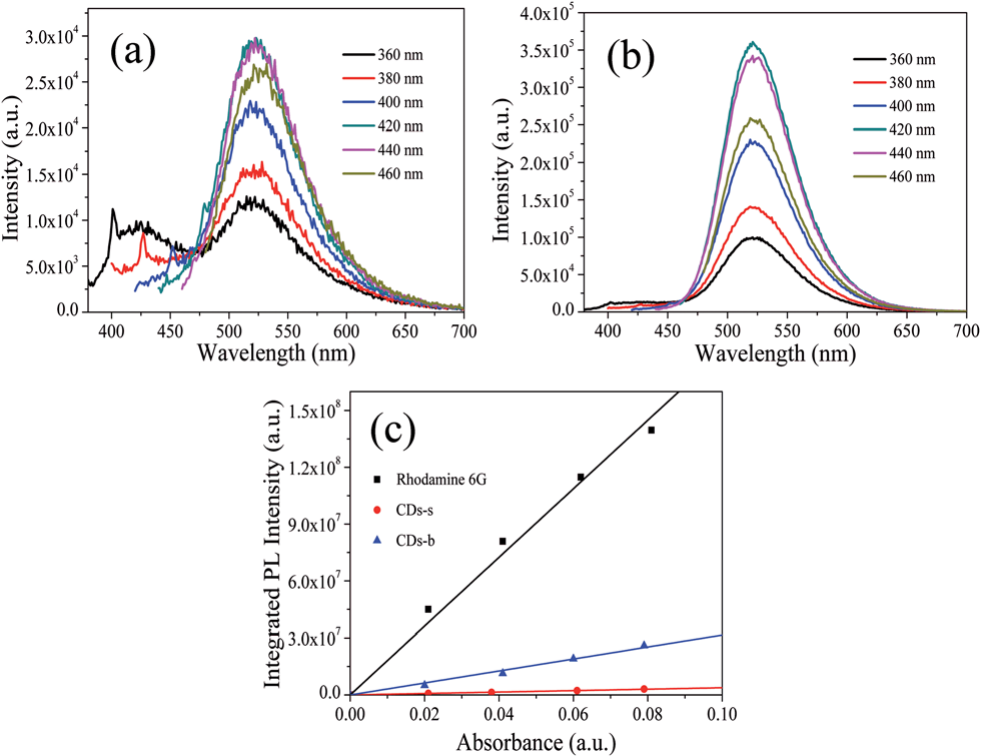
PL spectra at different excitation wavelengths of CDs-s (a), CDs-b (b) and QY measurement (c).

In order to confirm whether the green fluorescence of CDs-o originates from large-size CDs or small-size CDs, the CDs-o obtained were dialyzed in DMF. The solutions of CDs inside (CDs-s) and outside (CDs-b) the dialysis bag were obtained and the morphologies of CDs-s and CDs-b were investigated by TEM, as shown in Fig. 8. In Fig. 8(a), CDs-s are quasispherical and well dispersed, with little aggregation and an average size of 3.3 nm narrowly distributed from 2 to 4.5 nm. As shown in Fig. 8(b) for the TEM image of CDs-b, CDs-b are in quasispherical shape and show a wide size distribution with fractional aggregation, which is presumably connected with the hydrogen bond interaction arising from plentiful surface hydroxyl groups.^36^ The inset in Fig. 9 plots the size distribution histogram of CDs-b, which varies from 6 to 18 nm with average diameter of 11.9 nm, lager than that for most CDs ever reported.^37–40^ Unexpectedly, the size of CDs-b outside the dialysis bag is bigger than that of CDs-s inside the dialysis, which is different from what we commonly see. As we all know, dialysis bag is designed according to the molecular weight cut-off to separate the particles with different molecular mass. CDs-b and CDs-s may be two classes of CDs different from usually reported CDs which that are merely different in size. CDs-b and CDs-s have different properties in addition to their sizes, as will be discussed below. CDs-b outside the dialysis bag may own smaller molecular mass but lager size than CDs-s inside the dialysis, according to the shallower contrast of CDs-b than that of CDs-s in TEM images, which could be confirmed by ESI-MS. As seen from Fig. S1 (ESI†), no prominent signal is observed above 300 *m*/*z*, proving that the CDs-b outside the dialysis are mainly composed of small molecular fragments, which indicates that CDs-b own small molecular mass. As comparision, the prominent signal of CDs-s inside the dialysis is centered around 300 *m*/*z* and even week signal is also observed at 600 *m*/*z*, which indicates CDs-b outside the dialysis bag own smaller molecular mass than the CDs-s inside the dialysis.

Subsequently, the fluorescence properties of CDs-s and CDs-b were tested, and the results are shown in Fig. 9. The emission wavelength of CDs-s and CDs-b centers at 520 nm with excitation-independent nature. The QY of CDs-b is calculated to be 17.9%, as high as that of non-dialyzed CDs-o. Whereas the QY of CDs-s is only 2.20%, far below that of non-dialyzed CDs-o. Obviously, the green fluorescence of CDs-o mainly originates from the large sized green fluorescent CDs-b.

**Fig. 10:**
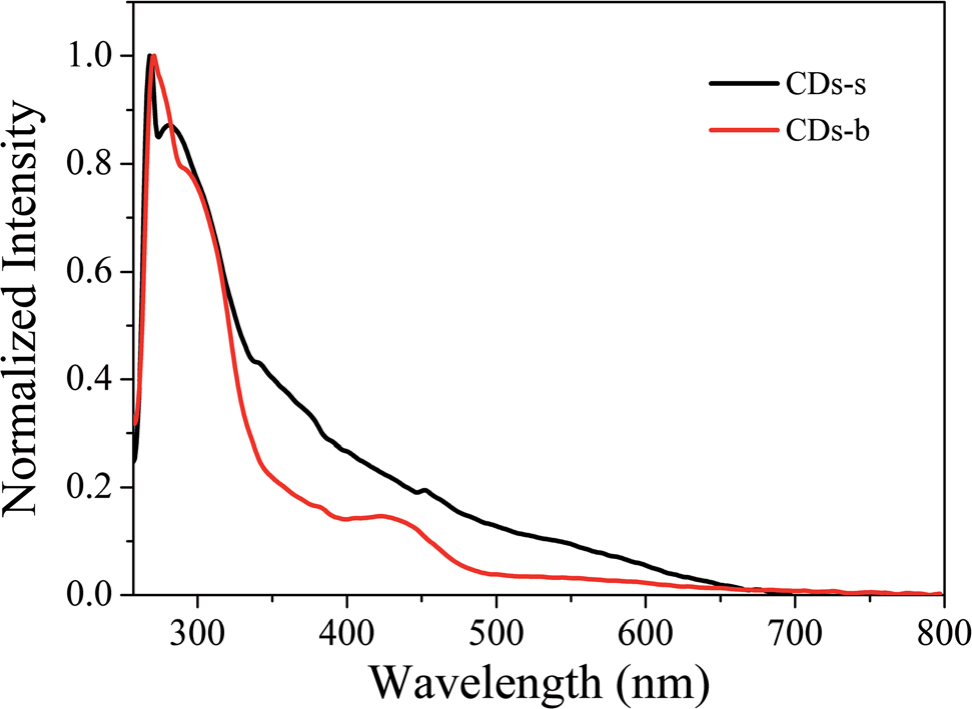
UV-vis absorption spectrum of CDs-s and CDs-b.

UV-vis absorption of CDs-s and CDs-b was measured further, as is shown in Fig. 10. For CDs-s, no significant absorption occurs after 400 nm, while for CDs-b an absorbance peak is observed at 420 nm, consistent with the UV-vis absorption spectra of non-dialysed CDs-o, which confirms that the green florescence of CDs-o originates mainly from large sized CDs-b and is closely associated with the absorption peak at 420 nm. Consequently, there exists large sp^2^-domain in large sized CDs-b.

The elemental analyses of CDs-o, CDs-s and CDs-b are summarized in Table S1 (ESI†). The element content of three classes of CDs is relatively close, indicating that the green fluorescence of CDs is not correlated with elemental content.

**Fig. 11:**
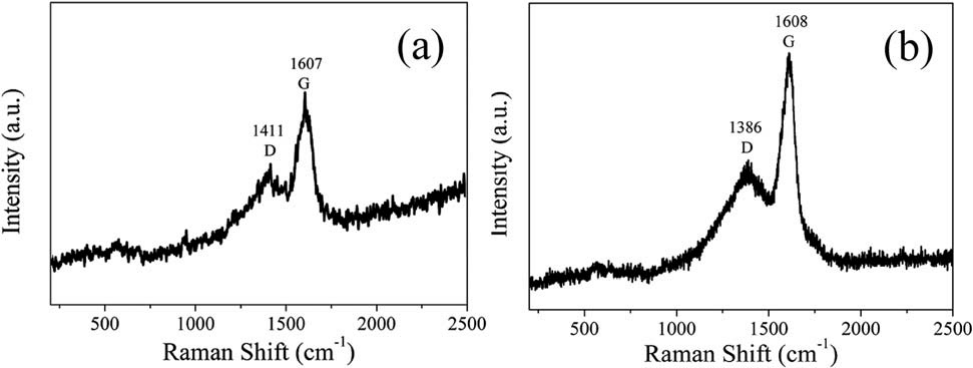
Raman spectrum of CDs-s (a) and CDs-b (b).

In order to analyze the structure of CDs-s and CDs-b further, Raman measurements were performed. As shown in Fig. 11, the Raman spectra of CDs-s and CDs-b both show obvious characteristic D band and G band at 1411, 1607 cm^−1^ and at 1386, 1608 cm^−1^ respectively. Remarkably, the intensity ratio *I*_G_/*I*_D_ of CDs-s and CDs-b is calculated to be 1.1 and 1.6, respectively, which indicates CDs-b have lager sp^2^-domain than CDs-s.

It is reported that DMF could be decomposed to dimethylamine and carbon monoxide at a higher temperature than its boiling point.^41^ It can be deduced that the dimethylamine formed at 180 °C during the solvothermal process reacts with the carboxylic groups of CDs intermediate (CDs-i), as can be seen from Fig. S2 (ESI†). In addition, the FT-IR (Fig. 5), high-resolution C1s spectra and elemental analysis of CDs-o reveal the existence of C–N (285.4 eV) (Fig. 6(b)) and the nitrogen content of 7.76% (Table S1†), further confirming the presence of nitrogen-doping process.^42^ As control experiments, two common solvents, water and ethanol, were also employed as reaction media for the reaction under the same conditions to obtain CDs-w and CDs-e. Elemental analysis indicates the products prepared in water and ethanol do not contain N element (Table S1†).

**Fig. 12:**
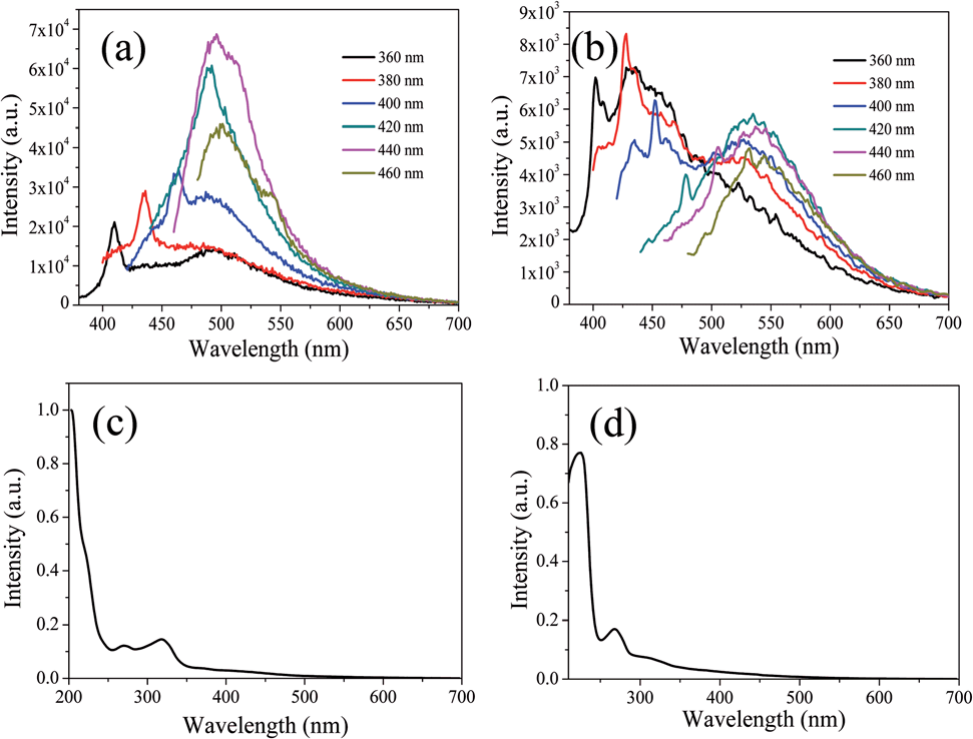
UV-vis absorption spectra, PL spectra at different excitation wavelengths of CDs-w (a and c) and CDs-e (b and d).

In order to further explain the role of DMF in helping CDs to form large conjugated sp^2^-domain and contributing to the green fluorescence emission of CDs, the optical properties of CDs-w and CDs-e were investigated, as shown in Fig. 12. The emission peaks of CDs-w and CDs-e under excitation at 360 nm locate at 490 nm and 430 nm respectively, which are lower than the emission peak centered at 520 nm for CDs-o obtained with DMF as solvent. With the increase of excitation wavelength, the emission intensity of CDs-w and CDs-e increases while the emission peak shifts to long wavelength gradually. Under the excitation wavelength of 440 nm and 420 nm respectively, the emission intensity reaches the maximum. As the excitation wavelength continues to increase, the emission intensity gradually decreases and the emission peak continues to shift to long wavelength. The excitation-dependent emission wavelength and intensity are common phenomena observed in carbon-based fluorescent materials. Such excitation-dependent PL behavior is related to the different surface states of CDs. There are multiple C- and O-containing functional groups on the surface of the produced CDs, resulting in various surface states with different energy levels and thus a series of emissive traps. Under different excitation wavelength, the corresponding surface state emissive trap is dominant, giving excitation-dependent PL.^43,44^ In order to further explore the structural differences of green emissive CDs-o from CDs-w and CDs-e, UV-vis absorption measurement was performed. As is shown in Fig. 12(c), the CDs-w obtained with water as solvent have two obvious absorption peaks at 270 and 318 nm, which are attributed to the π–π* transition of C=C bonds and n–π* transition of C=O bonds, respectively.^45^ The CDs-e prepared with ethanol as solvent exhibit two obvious absorption peaks at 224 and 268 nm (Fig. 12(d)), corresponding to the π–π* transition of the C=C bond and the n–π* transition of C=O, respectively.^46^ From these results, it can be seen that CDs prepared with either water or ethanol as solvent have no obvious absorption peaks after 400 nm, indicating that the obtained CDs do not have large conjugated sp^2^ structure, which proves that the green fluorescence of CDs-o is derived from the absorption peak around 420 nm. The QY of the CDs-w and CDs-e was calculated to be 0.15% and 0.62% respectively with rhodamine 6G as standard, far below that of the CDs prepared previously in our group, which are shown in Table S2 (ESI†). It may be due to the fact that pyrogallic acid used as carbon source in this work is not very active in chemical properties with merely hydroxyl as single functional group and without heteroatoms such as N, S, *etc.*, which endows the CDs prepared with fewer defect states and is not conducive to the improvement of QY. In contrast, most of the blue emissive CDs prepared previously in our group originated from the carbon sources with rich functional groups and heteroatoms, which makes the blue emissive CDs prepared be doped with heteroatoms on the carbon nuclei and surface more easily, thus is beneficial to the improvement of QY.

To investigate the morphology and size of CDs-w, TEM observation was conducted. As is shown in Fig. S3 (ESI†), CDs-w are quasispherical and well dispersed. The size of CDs-w is narrowly distributed from 2 to 5 nm with an average size of 3.5 nm, which is smaller than that of CDs-o.

In order to explore whether nitrogen doping can improve the QY and influence the fluorescence performance of CDs-w and CDs-e, 120 μL of ethylenediamine as nitrogen source was added under the same condition for reaction to obtain CDs-we and CDs-ee respectively. The optical properties of CDs-we and CDs-ee were investigated, as shown in Fig. S4 (ESI†). The emission peaks of CDs-we and CDs-ee under excitation at 360 nm locate at 501 nm and 439 nm respectively and show a certain red shift compared with the emission peaks of CDs-w and CDs-e, which may be due to the fact that the introduction of nitrogen increases the density of the electron cloud on the surface of the two classes of CDs and further causes the red shift of the emission peaks. With the increase of excitation wavelength, the emission intensity of CDs-we and CDs-ee increases while the emission peak shifts to long wavelength gradually. Under the excitation wavelength of 420 nm and 400 nm respectively, the emission intensity reaches the maximum. As the excitation wavelength continues to increase, the emission intensity gradually decreases and the emission peak continues to shift to long wavelength. Furthermore, UV-vis absorption measurement was performed. As is shown in Fig. S4(c),† CDs-we have three obvious absorption peaks at 224, 264 and 336 nm, which are attributed to the π–π* transition of C=C bonds, n–π* transition of C=O bonds and C=N bonds, respectively. CDs-ee exhibit two obvious absorption peaks at 265 and 305 nm (Fig. S4(d)†), corresponding to π–π* transition of the C=C bond and the n–π* transition of C=O, respectively. Further QY measurements were performed on CDs-we and CDs-ee and the QY of CDs-we and CDs-ee was calculated to be 0.24% and 1.27%, respectively. Compared with CDs-w and CDs-e, the QY of CDs-we and CDs-ee had a large improvement, which indicates that nitrogen doping is not only responsible for the red shift of the emission spectrum, but also beneficial to the QY improvement of CDs.^47^

CDs-o exhibit excellent solubility in many solvents, including acetone, acetonitrile, DMF, ethanol, methyl alcohol, and water. As shown in Fig. S5 (ESI†), CDs-o in different solvents exhibit different photoluminescence peaks under a single excitation wavelength. The emission peaks are observed at 526, 527, 520, 558, 568 and 581 nm in acetone, acetonitrile, DMF, ethanol, methyl alcohol, and water, respectively. In general, the emission peak shifts towards red as the solvent polarity increases. This solvent-dependent phenomenon is very similar to the “solvatochromism” in organic dyes. Many organic dyes' fluorescence emissions shift in different solvents. The solvatochromic effect is usually attributed to the intramolecular charge transfer.^48^ Furthermore, it can be clearly seen from Fig. S5(a–f)† that the fluorescence emission bands of CDs-o in any solvent do not shift with different excitation wavelengths. This strict excitation wavelength independence is distinctive in our CDs-o.

**Fig. 13:**
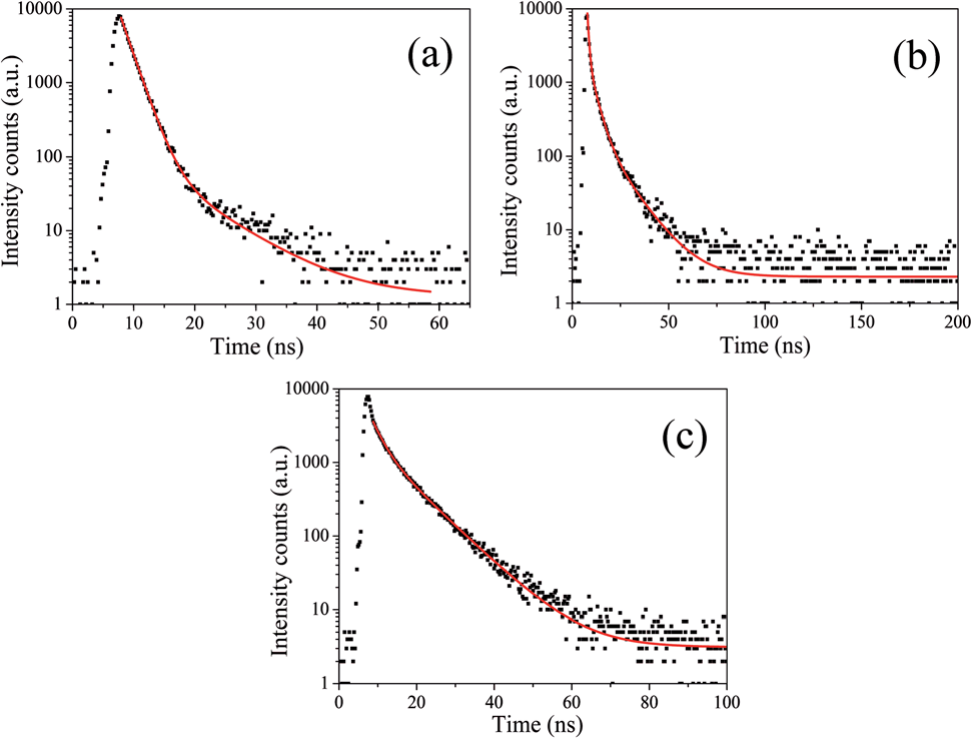
Time-resolved fluorescence decay curve of CDs-o (a), CDs-w (b) and CDs-e (c).

To acquire further insight into photoluminescence, the time-resolved fluorescence decay lifetime of CDs-o prepared with DMF as solvent, CDs-w prepared with water as solvent and CDs-e prepared with ethanol as solvent was measured under the excitation of 375 nm by using multidimensional time-correlated single photon counting (TCSPC) method, as seen in Fig. 13. The PL decay curves are analyzed by fitting the following eqn (1) and (2):1
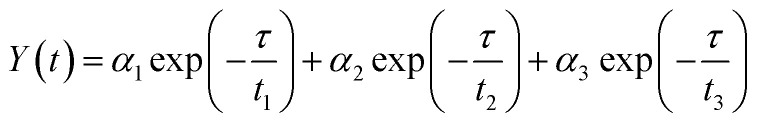
2
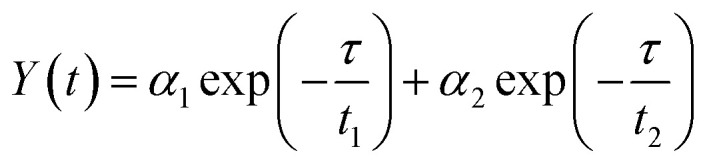
where, *α*_1_, *α*_2_ and *α*_3_ are the constants specifying the effect on decay with lifetimes *τ*_1_, *τ*_2_ and *τ*_3_. The average lifetime can be evaluated using the following relation:3
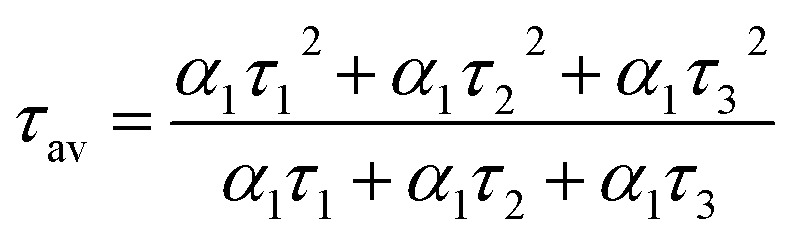
4
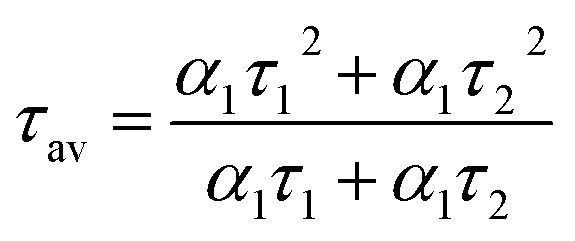


The mechanism of fluorescence is related to surface states and dark intrinsic state, which has been investigated by Wang's group.^49^ They found that the intrinsic state is attributed to the graphene core and its PL occurs with a dominant short time and low PL QY. The two radiative lifetimes *τ*_1_ and *τ*_2_ may be assigned to intrinsic recombination of initially populated core states and surface states, respectively.^50,51^ On the basis of our fit values in Table 1, CDs-o exhibit biexponential decay and the calculated average lifetime of the entire fluorescence decay process is 3.24 ns. The fractions of *τ*_1_ (short-lived component) is 93.43%, playing an dominating role in the radiative lifetime, which suggests that the fluorescence decay kinetics of these CDs-o is mainly caused by their core states and the green fluorescence of CDs-o originates from large conjugated sp^2^-domain in core. CDs-w and CDs-e exhibit tri- and biexponential decay with average lifetime of 7.88 and 8.00 ns, respectively. The fraction of *τ*_1_ for CDs-w and CDs-e is 35.83% and 28.14%, respectively, which suggests that the fluorescence decay kinetics of CDs-w and CDs-e are collectively caused by their core states and surface states and the latter plays a dominating role. It is well-known that diverse fluorophores or energy levels present in the samples are responsible for their multiple lifetimes.^52^ Therefore, it can be deduced that new energy levels are generated in CDs-w compared with CDs-o and CDs-e.

**Table 1:** Fitting parameters and average lifetime of CDs-o, CDs-w and CDs-e

	*τ*_1_ (ns)	*α*_1_ (%)	*τ*_2_ (ns)	*α*_2_ (%)	*τ*_3_ (ns)	*α*_3_ (%)	*τ*_av_ (ns)
CDs-o	1.78	93.43	7.90	6.57	—	—	3.24
CDs-w	0.74	35.83	3.57	43.79	11.55	20.38	7.88
CDs-e	2.63	28.14	8.64	71.86	—	—	8.00

### 3.5 Stability of CDs-o

**Fig. 14:**
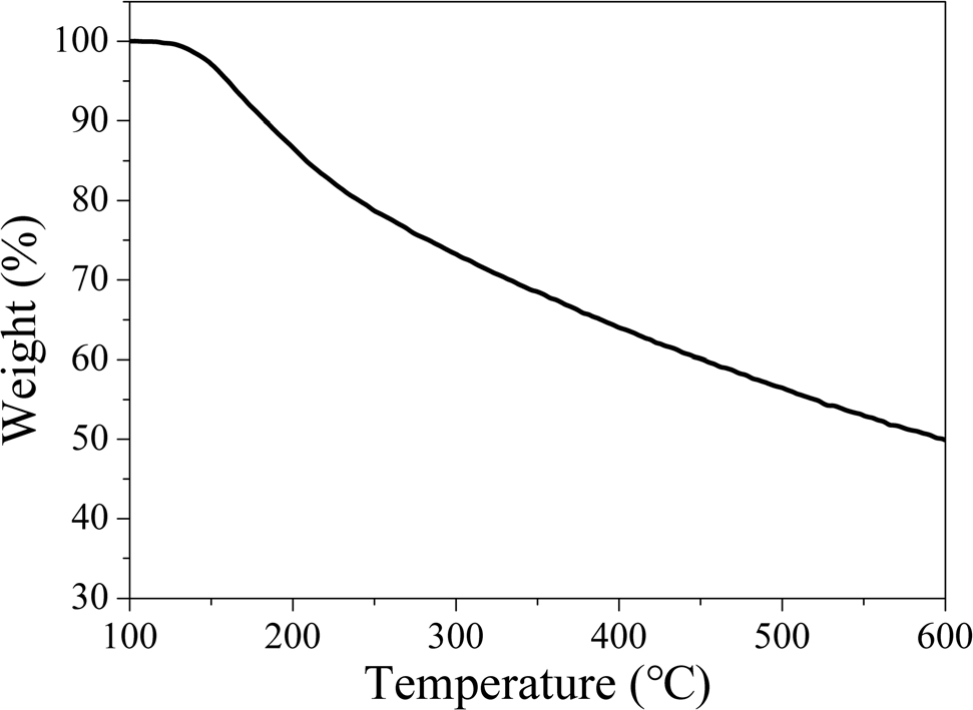
TG curves of CDs-o (N_2_ atmosphere, heating rate: 10 °C min^−1^).

Owing to the high working temperature of LED devices around 120 °C,^53^ the phosphors should have moderate thermostability. Therefore, TG analysis was conducted under nitrogen atmosphere to evaluate the thermostability of the samples. The TG curve in Fig. 14 reveals CDs-o have almost no weight loss at 100–130 °C, suggesting their sufficient thermostability in this temperature range.

Weight loss begins at 130 °C, with siginificant weight loss at 130–250 °C, which may be ascribed to the dehydration and release of the surface functional groups on CDs-o. Obviously, the thermostability of CDs-o can meet the requirements on LED phosphors.

Besides, the influence of ionic strength on the photoluminescence properties of CDs-o in aqueous solutions was investigated. As is shown in Fig. S6 (ESI†), the emission peak remains at 580 nm steadily under various concentrations of NaCl and the PL intensity of the CDs-o remains 84% of original strength in 1 mol L^−1^ NaCl, which demonstrates that the CDs-o possess good stability in high ionic-strength environments and can still play a part in a complex environment.

The effect of pH was also studied. As can be seen from Fig. S7 (ESI†), at lower and higher pH, CDs-o in aqueous solutions show low fluorescence intensity. Interestingly, when pH is at 3, CDs-o have strongest fluorescence intensity. This result may be caused by the carboxyl group and amino group on the surface of the CDs-o. The carboxyl group and amino group may be protonated in acidic solutions deprotonated in alkaline. Surely, the remarkable change of the stability of CDs-o with pH would not interference the application in LED.

Furthermore, the photostability of CDs-o in DMF solvent was evaluated using a UV light. As seen from Fig. S8 (ESI†), the UV light used in the test is harmful to CDs-o. The PL intensity of CDs-o decreases by less than 10% at first 5 min. Then, the decay rate of the PL intensity slows down. As a consequence of this, the PL intensity of CDs-o could be retained more than 40% after 60 min. The resistance against UV light of CDs-o can meet the requirements on LED phosphors on the whole.

### 3.6 Applications of CDs-o in LED

**Fig. 15:**
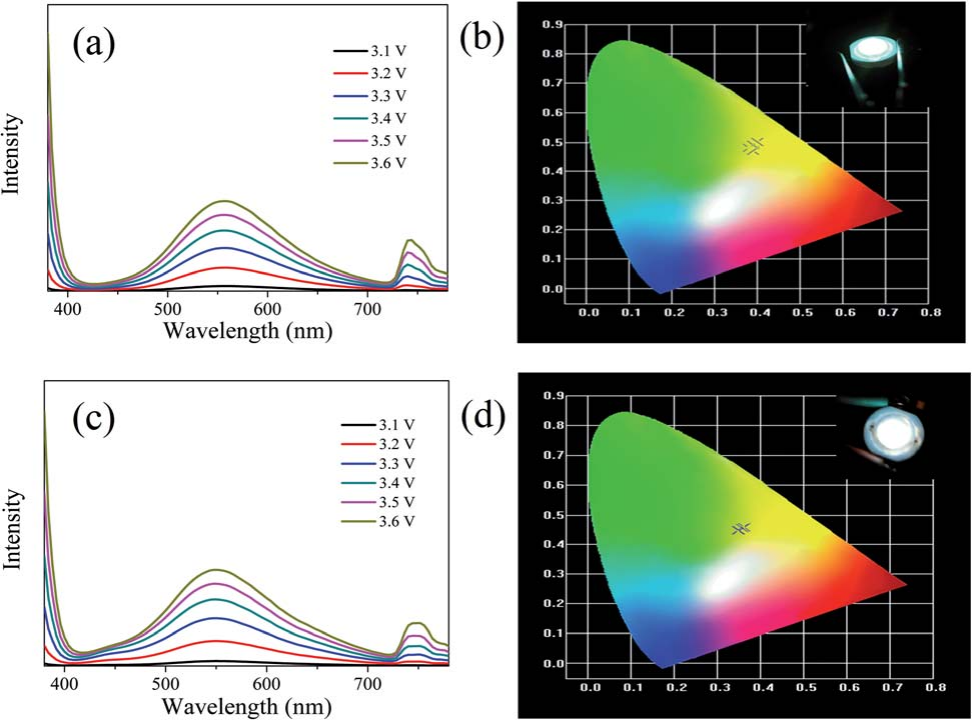
Emission spectra (a) and CIE chromaticity coordinates (b) of the white LED and emission spectra (c) and CIE chromaticity coordinates (d) of the white LED doped with several blue emissive CDs under different working currents (inset: digital photo of LEDs).

The LEDs based on CDs-o were fabricated, where CDs-o and KH-792 mixtures were coated on the solid-state lighting unit comprised of a UV chip with 365 nm emission. As shown in Fig. 15(a and b), the electroluminescent (EL) emission spectra of the device is composed of three parts. The peak before 400 nm originates from the UV chip. The emission peak around 740 nm is the defect peak of the UV chip. The emission peak around 560 nm is mainly derived from the green emissive CDs-o. There is a large degree of redshift of the emission peak of LED compared with the CDs-o in DMF, suggesting that a certain form of energy transfer arises between CDs-o and KH-792.^36^ Furthermore, the QY of the mixtures was measured to be 8.0%, which is lower than that of CDs-o in DMF solvent. This result further demonstrates the probability of energy transfer between CDs-o and KH-792. From the test results shown in Table 2, it can be seen that white lighting, though somewhat yellowish, is realized, and the CIE chromaticity coordinates and CCT of the device show a negligible change as the working voltage changes. The CCT of the LED is lower than that of most cool white LEDs with the CCT above 5000 K. The LED fabricated is suitable for the occasions needing warm white lighting such as indoor lighting.

**Table 2:** CCT and CIE chromaticity coordinates of the white LED under different working currents

Voltage/V	3.1	3.2	3.3	3.4	3.5	3.6
CCT/K	4285	4357	4435	4491	4503	4497
CIE coordinate	(0.39, 0.47)	(0.40, 0.50)	(0.39, 0.49)	(0.38, 0.48)	(0.38, 0.48)	(0.38, 0.48)

For further tuning the chromaticity, a small portion of blue emissive CDs with emission peak centered at 440 nm was doped along with the green emissive CDs into the LED. As shown in Fig. 15(c and d), compared with the LED above, a new weak emission peak centered at 440 nm appears, which is ascribed to the blue emissive CDs doped in the device. The test results in Table 3 show that CIE chromaticity coordinates of the white LED shift towards the pure white light direction and the CCT of the device becomes slightly higher owing to the introduction of blue emission.

**Table 3:** CCT and CIE chromaticity coordinates of the white LED doped with a small portion of blue emissive CDs under different working currents

Voltage/V	3.1	3.2	3.3	3.4	3.5	3.6
CCT/K	4811	5001	5120	5114	5037	4921
CIE coordinate	(0.36, 0.46)	(0.35, 0.45)	(0.35, 0.45)	(0.35, 0.45)	(0.35, 0.45)	(0.36, 0.45)

**Fig. 16:**
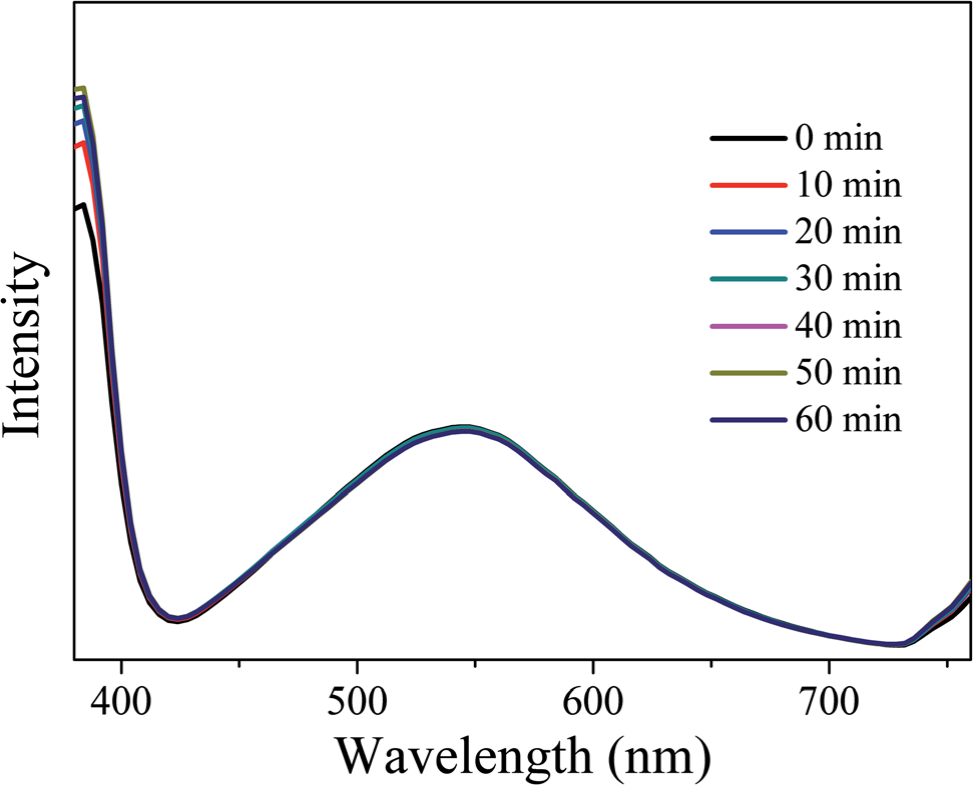
EL emission spectra of the LEDs at different working time intervals.

In order to investigate the optical stability of the resulting LEDs under long-term working time, emission spectra with different working time intervals at 3.5 V were measured. As shown in Fig. 16, the emission spectra remain stable and the intensity does not show obvious decay with the increase of working time, indicating that the obtained LEDs have excellent optical stability. Therefore, CDs-o show practical application value as phosphor in solid state lighting systems.

**Fig. 17:**
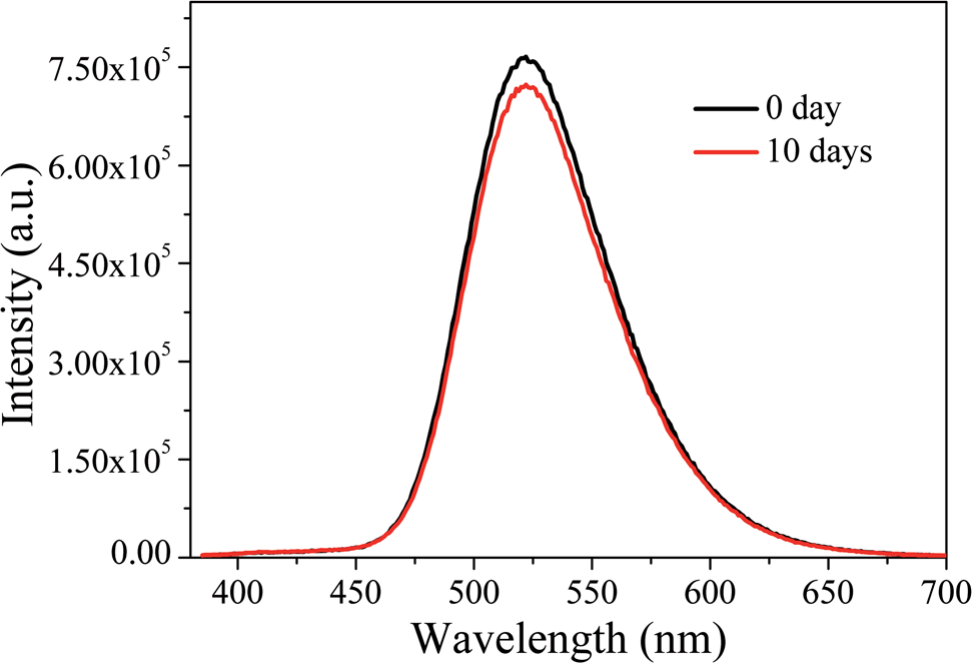
EL emission spectra of LEDs under long-term storage.

Subsequently, the optical stability of the LED under long-term storage was investigated. We selected the LEDs immediately fabricated and stored for ten days for optical performance tests. The results are shown in Fig. 17. It can be seen that after a 10 day-long period of storage, the emission spectra of LEDs do not change significantly, indicating that the LEDs have good stability under long-term storage.

Green emissive CDs are also expected to find their applications in other fields. Fig. 7 indicates that the CDs we prepared are able to emit light from 380 to 650 nm under the excitation from 360 to 450 nm, the wavelength ranges of both PL and excitation well fit the harvesting spectrum (480–650 nm) and unused light spectrum (380–480 nm) of the poly(3-hexylthiophene) : [6,6]-phenyl-C61-butyric acid methyl ester (P3HT : PCBM) based bulk heterojunction (BHJ) solar cell. When the composite containing CDs stands between the sun and the solar cell, the sunlight utilization efficiency of the latter would be improved accordingly. Besides, the highly N-doped CDs (N-CDs) may be used for heavy metal ion and biomolecule detection because the doped N atoms would facilitate the coordination interaction and further enhance the selectivity to the analyte.^54^ In addition, longer wavelength emission usually allows a deeper penetration of photon into cells and tissues to form a more comprehensive imaging, which is favorable for bioimaging. So our green emissive CDs with longer wavelength emissions centered at 520 nm can be used for bioimaging owing to their good water-solubility and their green fluorescence can not be interferenced with blue fluorescence emitted from some cells and tissues under ultraviolet light.^55^

## 4 Conclusions

Nitrogen-doped fluorescent CDs were obtained with pyrogallic acid as precursor and DMF as solvent by solvothermal method. The QY of the CDs was calculated to be 16.8%. Under the excitation of ultraviolet light, the CDs emit bright green fluorescence, owing to their large average particle size of 11.9 nm and large conjugated sp^2^-domain. Comparision of the CDs prepared with water and ethanol as solvent with the CDs prepared with DMF as solvent gives evidence that DMF favors the formation of large conjugated sp^2^-domain in CDs. The presence of N element in the CDs indicates that DMF not only acts as solvent providing a place for the reaction, but also participates in the formation of CDs. A series of characterizations propose that the green fluorescence of the CDs is derived from the carbon core and related to the large conjugated sp^2^-domain in the CDs. The CDs is moderately thermally stable to meet the thermostability requirements of LED phosphor. Further, white light LEDs were fabricated by using the CDs as phosphor and a UV chip. When the driving voltage varies between 3.1 and 3.6 V, the CCT of the LED lies in the range of 4285–4503 K, which belongs to warm white light and suitable for the occasions needing warm white lighting such as indoor lighting.

## Conflicts of interest

There are no conflicts to declare.

## Supplementary Material

RA-008-C8RA02226G-s001
