# Supplementary material for: The synthesis of green fluorescent carbon dots for warm white LEDs
Source: RSC Adv. 2018 May 29;8(35):19585–95. doi: 10.1039/c8ra02226g (PMC9080646; doi:10.1039/c8ra02226g)
Supplement: RA-008-C8RA02226G-s001 [file RA-008-C8RA02226G-s001.pdf]

Electronic Supplementary Material (ESI) for RSC Advances.
This journal is © The Royal Society of Chemistry 2018

# Electronic Supplementary Information

## The Synthesis of Green Fluorescent Carbon Dots for Warm White LEDs

Du Qin [a,b], Zheng Jingxia [a,c], Wang Junli [a,c], Yang Yongzhen* [a,c], Liu Xuguang* [a,d]

[a]Key Laboratory of Interface Science and Engineering in Advanced Materials (Taiyuan University of Technology), Ministry of Education, Taiyuan 030024, China

[b]College of Chemistry and Chemical Engineering, Taiyuan University of Technology, Taiyuan 030024, China

[c]Research Center of Advanced Materials Science and Technology, Taiyuan University of Technology, Taiyuan 030024, China

[d]College of Advanced Materials Science and Engineering, Taiyuan University of Technology, Taiyuan 030024, China

*Corresponding authors: Email: liuxuguang@tyut.edu.cn (Liu Xuguang), yyztyut@126.com (Yang Yongzhen).

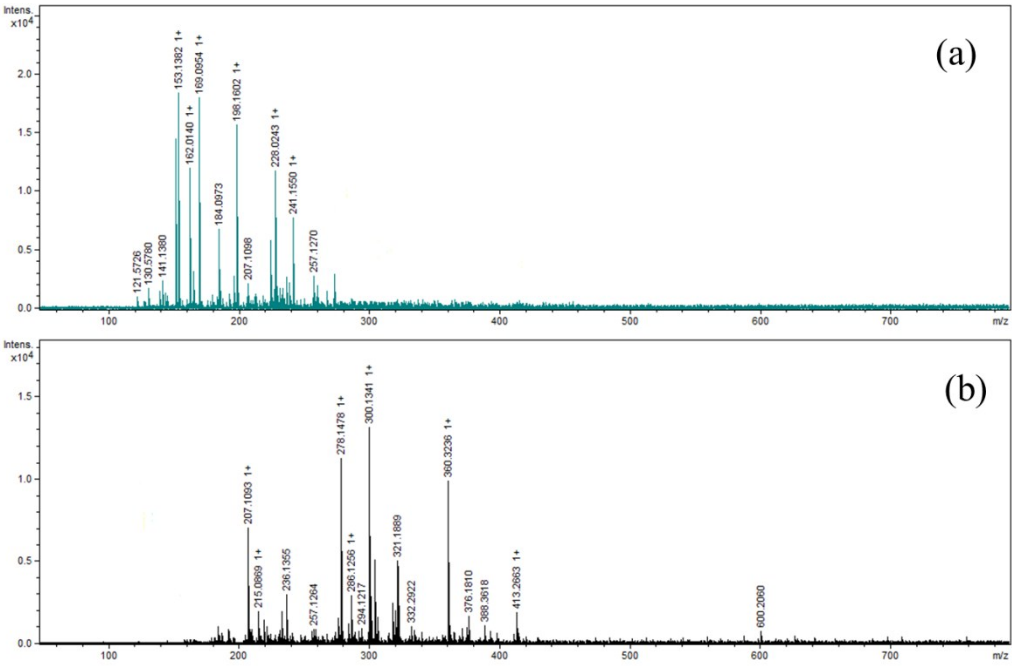


Figure S1 ESI-MS spectra of CDs-b (a) and CDs-s (b) in the region of 0–800 m/z.

Table S1 Elemental composition of CDs-o, CDs-s, CDs-b, CDs-w and CDs-e

| | C (wt%) | H (wt%) | N (wt%) | O* (wt%) |
|---|---|---|---|---|
| CDs-o | 52.80 | 5.44 | 7.76 | 34.00 |
| CDs-s | 55.00 | 5.60 | 6.67 | 32.73 |
| CDs-b | 53.24 | 5.63 | 7.08 | 34.05 |
| CDs-w | 55.51 | 4.97 | 0 | 39.52 |
| CDs-e | 56.52 | 5.08 | 0 | 38.40 |

*By difference.

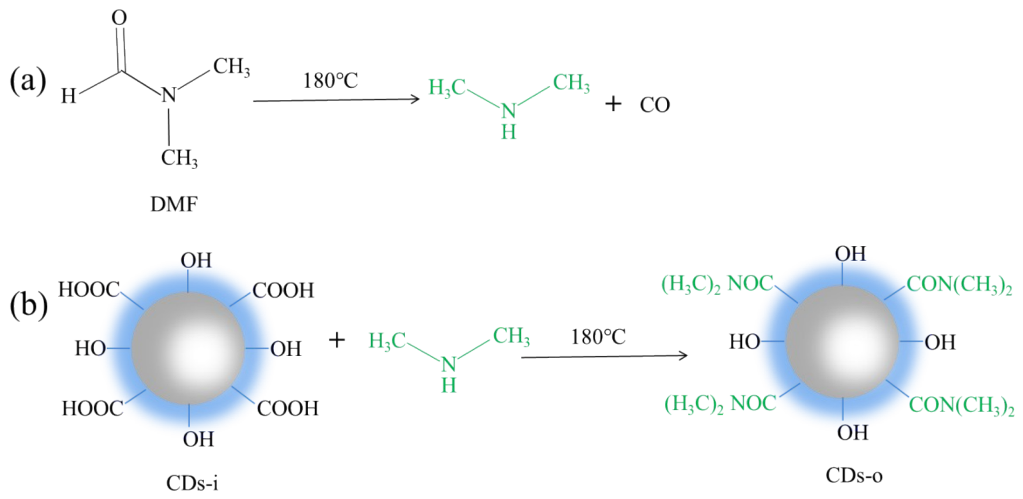


Figure S2 (a) Decomposition of DMF at high temperature. (b) Schematic illustration of the strategy for preparation of CDs-o.

Table S2 The QY of blue emissive CDs prepared by our group

| materials | solvent | emission peak (nm) | approach | QY | references |
|---|---|---|---|---|---|
| citric acid, KH-792 | water | 448 | Hydrothermal method | 57.3% | [1] |
| citric acid, ethylenediamine | water | 444 | Hydrothermal method | 67.06% | [2] |
| citric acid, ammonia solution | water | 440 | Hydrothermal method | 35.4% | [3] |
| citric acid, L-cysteine | water | 420 | Hydrothermal method | 67.41% | [4] |
| ammonium citrate, phosphates | water | 450 | Hydrothermal method | 62% | [5] |

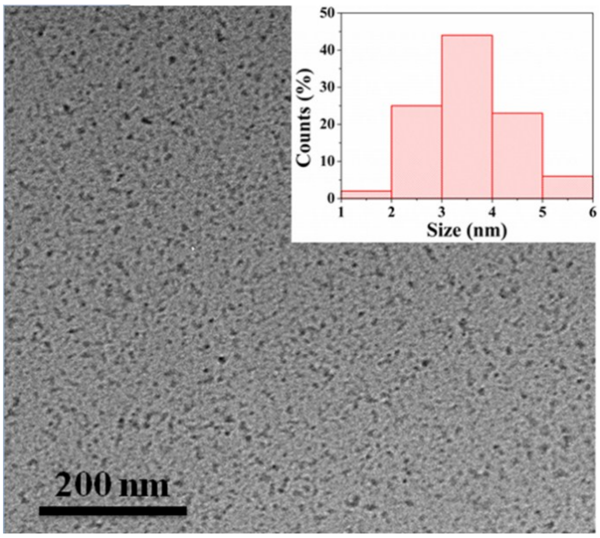


Figure S3 TEM images of CDs-w (inset is the size distribution).

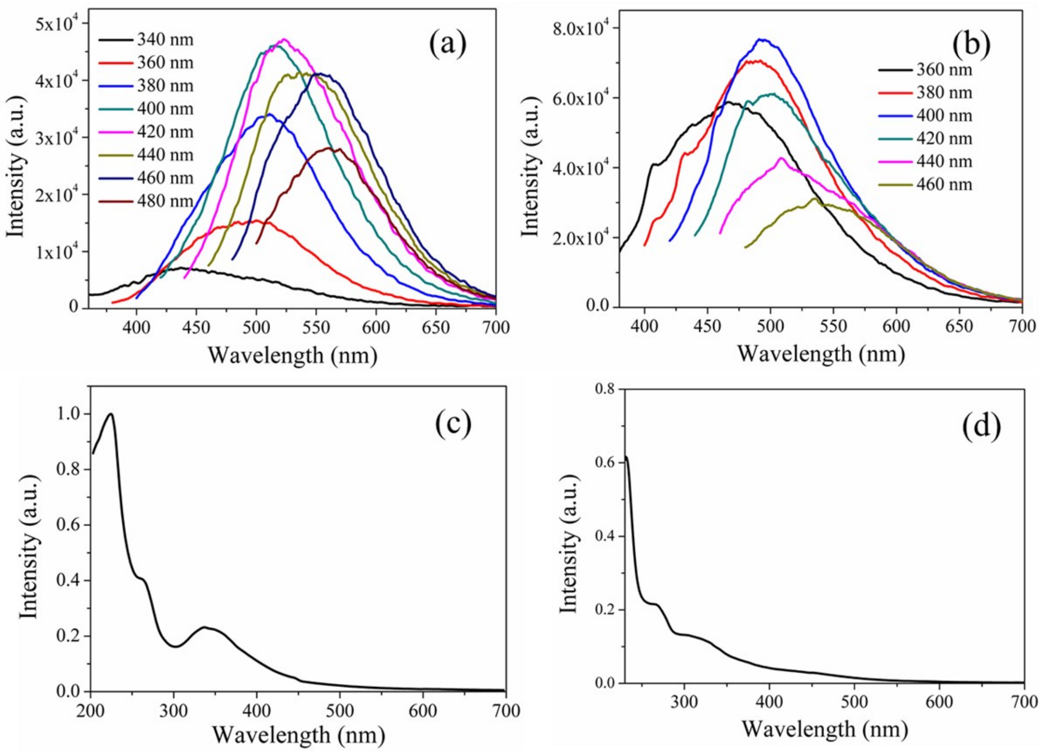


Figure S4 UV-vis absorption spectra, PL spectra at different excitation wavelengths of CDs-we (a, c) and CDs-ee (b, d).

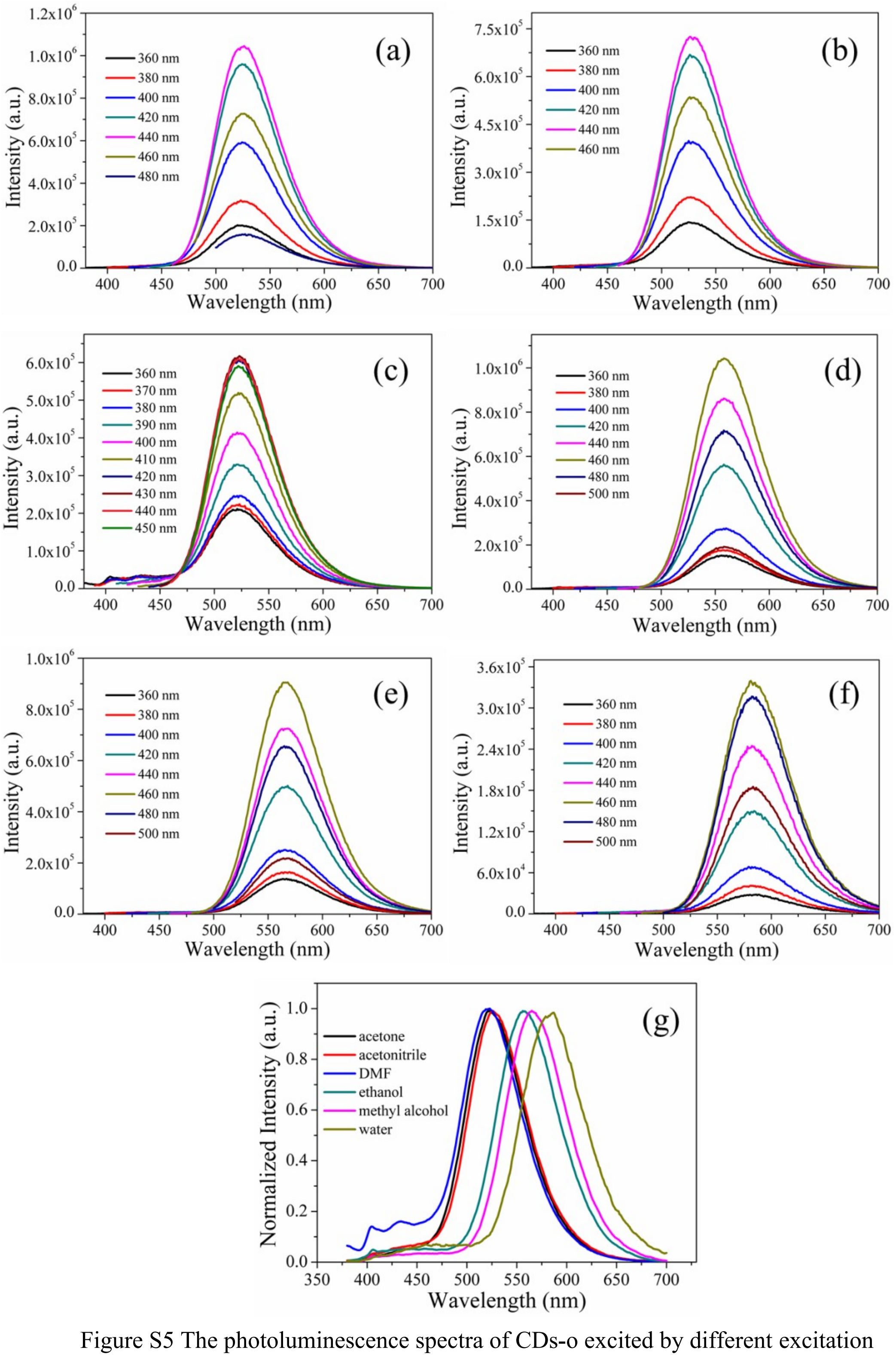


Figure S5 The photoluminescence spectra of CDs-o excited by different excitation wavelengths in solvents of acetone (a), acetonitrile (b), DMF (c), ethanol (d), methyl alcohol (e), water (f), and the photoluminescence spectra of the same CDs in 6 solvents excited by 360 nm light (g).

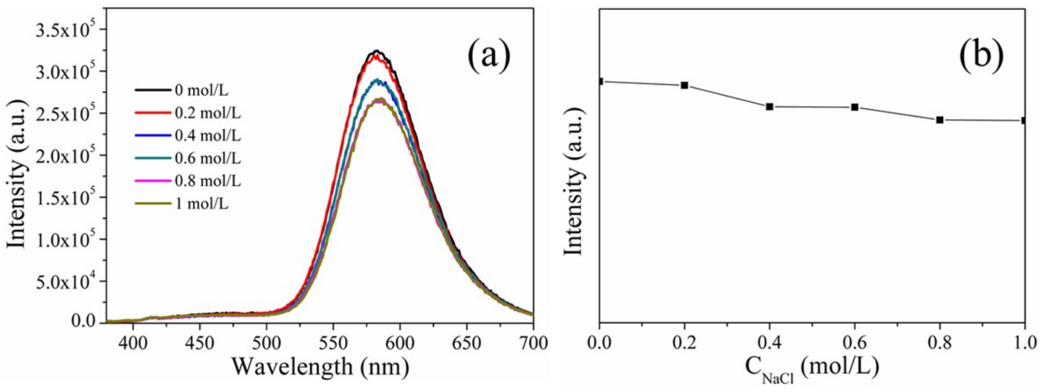


Figure S6 The photoluminescence spectra of CDs-o aqueous solutions (a) and PL intensity (b) (regulated by concentrations of NaCl from 0 to 1 mol/L).

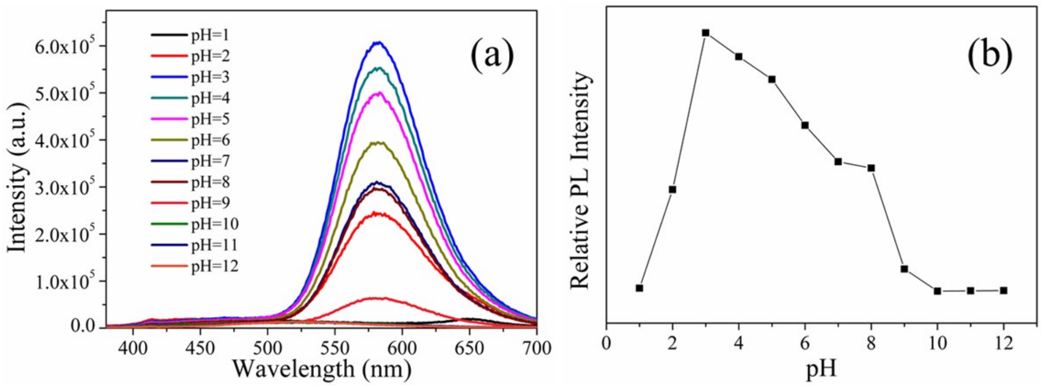


Figure S7 The photoluminescence spectra (a) and relative PL intensity of CDs-o aqueous solution with the increase of pH from 1 to 12.

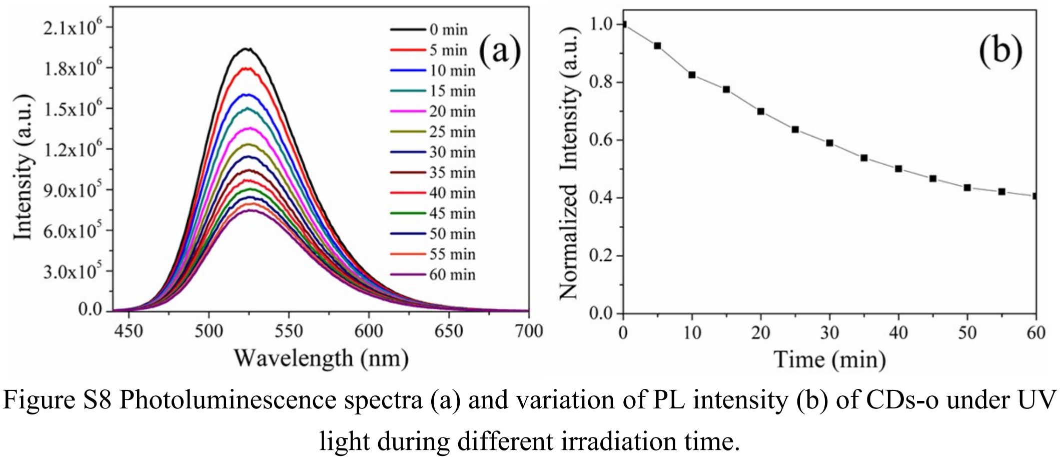


Figure S8 Photoluminescence spectra (a) and variation of PL intensity (b) of CDs-o under UV light during different irradiation time.

## References


[1] F. Zhang, X. T. Feng, Y. Zhang, L. P. Yan, Y. Z. Yang and X. G. Liu. *Nanoscale*, 2016, **8**, 8618–8632.

[2] Y. L. Wang, Y. Q. Zhao, Y. Zhang, F. Zhang, X. T. Feng, L. Chen, Y. Z. Yang and X. G. Liu. *RSC Advances*, 2016, **6**, 38761–38768.

[3] Y. Zhang, P. P. Cui, F. Zhang, X. T. Feng, Y. L. Wang, Y. Z. Yang and X. G. Liu. *Talanta*, 2016, **152**, 288–300.

[4] Y. L Wang., Y. Q. Zhao, F. Zhang, L. Chen, Y. Z. Yang and X. G. Liu. *New Journal of Chemistry*, 2016, **40**, 8710–8716.

[5] F. Zhang, Y. L. Wang, Y. Q. Miao, Y. H. He, Y. Z. Yang and X. G. Liu. *Applied Physics Letters*, 2016, **109**, 083103–083107.
